# Electroless Palladium-Coated Polymer Scaffolds for Electrical Stimulation of Osteoblast-Like Saos-2 Cells

**DOI:** 10.3390/ijms22020528

**Published:** 2021-01-07

**Authors:** Oriol Careta, Asier Salicio-Paz, Eva Pellicer, Elena Ibáñez, Jordina Fornell, Eva García-Lecina, Jordi Sort, Carme Nogués

**Affiliations:** 1 Departament de Biologia Cel·lular, Fisiologia i Immunologia, Universitat Autònoma de Barcelona, E-08193 Bellaterra (Cerdanyola del Vallès), Spain; oriol.careta@uab.cat (O.C.); elena.ibanez@uab.cat (E.I.); 2 CIDETEC, Basque Research and Technology Alliance (BRTA), Paseo Miramón 196, E-20014 Donostia-San Sebastián, Spain; asalicio@cidetec.es (A.S.-P.); egarcia@cidetec.es (E.G.-L.); 3 Departament de Física, Universitat Autònoma de Barcelona, E-08193 Bellaterra (Cerdanyola del Vallès), Spain; eva.pellicer@uab.cat (E.P.); jordi.sort@uab.cat (J.S.); 4 Institució Catalana de Recerca i Estudis Avançats (ICREA), Pg. Lluís Companys 23, E-08180 Barcelona, Spain

**Keywords:** electrical stimulation, osteoblast, Pd-coated polyurethane scaffold, gene expression, differentiation

## Abstract

Three-dimensional porous scaffolds offer some advantages over conventional treatments for bone tissue engineering. Amongst all non-bioresorbable scaffolds, biocompatible metallic scaffolds are preferred over ceramic and polymeric scaffolds, as they can be used as electrodes with different electric field intensities (or voltages) for electric stimulation (ES). In the present work we have used a palladium-coated polymeric scaffold, generated by electroless deposition, as a bipolar electrode to electrically stimulate human osteoblast-like Saos-2 cells. Cells grown on palladium-coated polyurethane foams under ES presented higher proliferation than cells grown on foams without ES for up to 14 days. In addition, cells grown in both conditions were well adhered, with a flat appearance and a typical actin cytoskeleton distribution. However, after 28 days in culture, cells without ES were filling the entire structure, while cells under ES appeared rounded and not well adhered, a sign of cell death onset. Regarding osteoblast differentiation, ES seems to enhance the expression of early expressed genes. The results suggest that palladium-coated polyurethane foams may be good candidates for osteoblast scaffolds and demonstrate that ES enhances osteoblast proliferation up to 14 days and upregulate expression genes related to extracellular matrix formation.

## 1. Introduction

The use of three-dimensional (3D) porous scaffolds in bone tissue engineering has been introduced as a promising alternative to conventional treatment options (i.e., autografting, allografting and xenografting) to promote bone healing and regeneration of relatively large non-healing bone defects, as it offers some advantages over conventional treatments (i.e., avoidance of donor site morbidity, high supply availability or reduced immune rejection) [1,2,3]. 3D porous scaffolds must be compliant with the following requirements to be used in bone tissue engineering: (i) open and interconnected pore network to allow cell nutrition, proliferation and migration for tissue vascularization and formation of new tissue, (ii) in vivo vascularization, (iii) biocompatibility and bioactivity/biodegradability (iv) similar mechanical properties to those of the replaced tissue (v) corrosion resistance and (vi) ease of fabrication [1,4,5].

Among the non-bioresorbable scaffolds, ceramics are the most widely studied materials as they offer a combination of high fracture toughness with good biocompatibility [6]. However, their associated brittle behavior and the typically reported high stiffness may hamper their application in specific cases where a certain degree of elasticity is required. For example, an implantable bone scaffold should not be too stiff so as to limit the stress shielding effect and avoid bone resorption and scaffold loosening, but should offer enough mechanical support and long-term stability for any daily activity [7]. Polymers are also considered as potential biomaterials for scaffolds due to some of their characteristics like high ductility and easy processability [8]. However, polymers also suffer from some drawbacks such as an exceedingly low elastic modulus and, in some cases, poor corrosion resistance. In this framework, biocompatible metallic scaffolds offer two main advantages over ceramic and polymeric scaffolds: (i) their mechanical properties are more similar to those of the human bone and (ii) they are intrinsically conductive, which makes them interesting candidates for electrical stimulation. However, metals typically suffer from corrosion in bodily fluids, leading to eventual ions release into the medium. For this reason, the use of alloying elements which upon dissolution release non-cytotoxic ions is of utmost importance. Several factors have to be considered like the chemical nature of the released ion (i.e., its oxidation state and complexation degree with the medium species) and dose.

For decades, the application of an external electric field has been studied and successfully used in clinical practice to stimulate bone healing [3]. It has been shown that electrical stimulation (ES) modifies osteoblast activities including adhesion, proliferation, nodule formation, gene expression, protein synthesis, and bone formation markers [9].

Various materials have been used as electrodes with different electric field intensities (or voltages) for electric stimulation in osteogenic functions. For instance, conductive polymers such as polypyrrole have been widely explored for bone stimulation [2,10,11,12,13,14]. Another approach to provide electrical conductivity to ceramic or polymeric scaffolds is based on the incorporation of carbon nanotubes (CNT) [15,16,17]. Mata et al. [17] proved that ES clearly induced cell growth and differentiation on the electroconductive CNT/Hydroxyapatite/Glass designed composite material. Vila et al. [16] demonstrated that bone cell metabolism was stimulated and mitochondrial activity was increased up to seven times in the presence of CNT/mesoporous silica composites under electrical stimulus. In turn, 3D porous and electrically active TiO_2−x_ ceramic scaffolds were produced by the foam replica method but, to the best of our knowledge, the in vitro cell response to ES has not been studied so far [1]. On the other hand, piezoelectric materials have also been used to stimulate electrically excitable cells (e.g., cardiomyocytes, skeletal myotubes, osteoblasts, neural cells) to modulate their proliferation and differentiation [18,19,20,21,22]. Contrary to the above materials, piezoelectric ones are able to perform cell stimulation in the absence of any external stimuli.

Fewer studies have been focused on the use of metallic scaffolds. Although these materials exhibit high electrical conductivity and ease of processability, their use has been precluded due to the cytotoxic effects of some metallic materials, as aforementioned. However, successful results have been obtained in electrically stimulated biocompatible TiO_2_ nanotubes grown on Ti substrates [23]. The authors showed that electric fields triggered osteogenic induction of mesenchymal stem cells and they also stimulated the formation of plasma membrane protrusions and the transport of connexin 43 to these protrusions. No cytotoxic effects were observed with electrically stimulated fibronectin-coated gold nanoparticles [24], whose unique properties were able to induce differentiation of stem cells into the mesodermal lineage. Importantly, electric fields can be created through direct ohmic contact of the material with the hardwires or wirelessly. The latter is referred to as bipolar electrochemistry [25] and consists of placing hardwired electrodes in the cell culture medium containing the material without direct ohmic contact with it. The possibility to electrostimulate cells by wirelessly controlling an implanted material has obvious advantages, as it is much less invasive. Very recently, the use of conducting polymers based on polypyrrole for contactless electrostimulation has been reported [26].

In this study, we have used a palladium (Pd)-based scaffold as a bipolar electrode to electrically stimulate human osteoblast-like Saos-2 cells. Although alloys that contain Pd as the major component have not been used as scaffolds in the orthopaedic field so far, high-content Pd alloys have been extensively used for dental prostheses and, more recently, for temporary and permanently implantable devices [27,28]. Despite concerns about the harmful effects of low-level corrosion products during biomedical use, all current evidence suggests that Pd alloys are safe [27,28,29]. Pd-based scaffolds were produced by a cost-efficient electroless process where polyurethane (PU) foams were used as templates. The ultimate aim of our work is to combine the invaluable properties of Pd-based materials (in terms of electrical conductivity, mechanical performance and biocompatibility) with the above-mentioned advantages of highly porous 3D scaffolds to eventually improve bone healing. With this goal in mind, Pd-metallized PU foams were used in the present work as bipolar electrodes to study the effect of electrical stimulation on the proliferation and differentiation of osteoblast cells grown on them.

## 2. Materials and Methods

### 2.1. Pd-Coated PU Foam Synthesis

Palladium metallization sequence of PU foams was carried out in glass vessels with a total volume of 500 cm^3^. In all the cases, temperature was controlled by a PT 1000 probe attached to the magnetic stirrer (Basic, IKA). PU templates of arbitrary shape were first cleaned with ethanol followed by alkaline soup and rinsed afterwards. To catalytically activate the PU foams for the subsequent metallization process, a two-step process, in which a metallic seeding was carried out (sensitization) followed by an acidic rinse stage (accelerator). In the former, colloidal palladium nuclei surrounded by a divalent tin shell were deposited over the PU surface. In the latter, the excess of divalent tin was eliminated so the palladium nuclei act as nucleation sites for the upcoming electroless process. In this study, the PU templates were sensitized with a commercial Pd/Sn colloidal solution (Neolink, Atotech Deutschland) at 40 ± 1 °C and pH = 1 for 15 min without stirring. Then, the samples were immersed in an accelerator solution (Adhemax, Atotech Deutschland) working at 48 ± 1 °C, pH = 1 and ω = 200 rpm for 2 min. Activated PU templates were transferred to a hypophosphite based electroless palladium electrolyte (Talon 3.0, Uyemura). The Pd solution worked at 50 ± 1 °C and pH 9.5. NH_4_OH solution was used for pH adjustment. Metallization time was fixed at 20 min. Palladium salts, sodium hypophosphite and complexing agents of the electroless palladium electrolyte were replenished accordingly after each metallization process. Note that the electroless coating consists of Pd metal with 6 wt.% P. For simplicity, we refer to it as Pd coating or metallization. The Pd-metallized PU foams were rinsed and gently air blown. Remaining water kept inside the porous structure was eliminated by drying the samples in an electric oven at 60 °C for 6 h.

### 2.2. Scanning Electron Microscopy Analysis

The morphology, composition and thickness of the Pd-coated PU foams was assessed by scanning electron microscopy (SEM, Merlin FE-SEM, Zeiss, Oberkochen, Germany) equipped with an energy-dispersive X-ray spectroscopy (EDX) detector. Secondary electrons were used for imaging purposes at an operating voltage of 5 keV. Compositional analyses were carried out at 15 keV.

### 2.3. X-ray Diffraction Analysis

X-ray diffraction (XRD, Philips X’Pert powder diffractometer) was used to assess the crystallographic structure of the Pd-coated PU foams. Sample preparation was required for XRD characterization. Specifically, the Pd-coated PU foams were smashed to obtain a powder-like morphology. Rietveld refinement was performed using the HighScore Plus software and crystallite size, microstrain and cell parameter values were calculated.

### 2.4. Cell Culture

Human osteoblast-like Saos-2 cells (ATCC HTB-85) were cultured in Dulbecco’s Modified Eagles Medium (DMEM) (Gibco, ThermoFisher Scientific, Waltham, MA, USA) supplemented with 10% foetal bovine serum (Gibco), at 37 °C and 5% CO_2_. Pd-coated PU foams of approximately 0.2 cm^3^ (1 × 1 × 0.2 cm) were sterilized with absolute ethanol and individually introduced into the wells of a 4-well plate. Then, 200,000 Saos-2 cells were seeded into each well. After 72 h of culture, cell-containing Pd-coated PU foams were transferred into a 6-well plate and cultured under standard conditions (SC) or electrical stimulation (ES). In parallel, the same number of cells was seeded directly onto a glass coverslip placed in a 6-well plate in the absence of Pd-coated PU foams (control) and cultured under SC or ES. The glass coverslip or the Pd-coated PU foam were placed in the middle of the culture plate at 75 mm distance of the platinum electrodes, without direct ohmic contact.

### 2.5. Construction and Use of an Electrical Stimulation Chamber

Two stimulation chambers were constructed as described by Leppik et al. [30] and used for ES of Saos-2 cells during culture. Briefly, the design consisted of platinum electrodes secured to the top lid (from now on, ES lid) of a cell culture chamber that fits on a standard 6-well cell-culture plate, allowing for easy handling and sterilization and minimal medium evaporation. Electrodes were bent into an L-shape and two electrodes were firmly fixed to the lid and sealed with silicone glue precisely 25 mm apart, to fit inside each culture well of standard 6-well cell culture plates. The tips of the electrodes, which protrude from the lid, were soldered to silver-coated copper wires in a parallel circuit and connected to a standard electric power supply (B2962A 6.5 Digit Low Noise Power Source, Keysight technologies, Santa Rosa, CA, USA) by means of banana connectors. For sterilization, the ES lid was immersed in 70% ethanol for 30 min and then exposed to UV light for 3 min in a UV chamber inside the flow hood. After 3 days of culture, the standard lids of the culture plates were exchanged for the ES lids and connected to the power supply. Cells were exposed daily to an arbitrary sinusoid function of 2.5 V amplitude and 200 Hz frequency for 1 h. After each ES exposure time, the ES lids were exchanged again for the original lids and cultures were maintained under standard conditions until the next stimulation time. The ES lids were cleaned and sterilized to be reused the next day.

### 2.6. Cell Proliferation Assay

Cell proliferation was assessed with the Alamar Blue assay (Thermo Fisher Scientific) at 3, 7, 14 and 28 days of culture. First, the culture media was replaced by 1 mL of DMEM containing 10% Alamar Blue dye, and cells were incubated at 37 °C in the dark for 4 h. Then, the Alamar Blue solution was removed, and its fluorescence was measured at 585 nm wavelength after excitation at 560 nm on a Varian Cary Eclipse Fluorimeter (Agilent, Santa Clara, CA, USA). Fresh medium was added to cell cultures, which were maintained in standard conditions until the next assay.

### 2.7. SEM Analysis of Cell Morphology

Cell morphology and adhesion to Pd-coated PU foams was assessed by SEM. Cells were washed in 0.1 M cacodylate buffer saline (CBS), fixed in 2.5% glutaraldehyde in 0.1 M CBS for 45 min at RT and rinsed again twice in CBS. Cell dehydration was performed in a series of ethanol washes (50, 70, 90 and twice 100%) for 8 min each. Finally, samples were dried using hexamethyldisilane (HMDS; Electron Microscopy Sciences, Hatfield, PA, USA) for 15 min. Samples were mounted on special stubs and analyzed using a SEM Merlin (Zeiss) equipped with EDX detector.

### 2.8. Actin Staining

For further cell adhesion examination, cell cultures were rinsed twice with PBS and fixed with 4% paraformaldehyde in PBS for 30 min at RT, then rinsed twice in PBS. Cells were permeabilized with 0.1% triton X-100 in PBS for 15 min at RT. Actin filaments were stained by incubating the samples with Alexa Fluor 594-conjugated phalloidin (Invitrogen, ThermoFisher Scientific, Waltham, MA, USA), and the cell nucleus was stained with Hoechst 33,258 (Sigma-Aldrich, Saint Louis, MO, USA) for 15 min at RT in the dark. Saos-2 actin distribution was observed under a confocal microscope (Confocal TCS SP5, Leica, Wetzlar, Germany).

### 2.9. Real-Time Quantitative PCR

The expression of osteogenic marker genes encoding alkaline phosphatase (ALPL), osteocalcin (BGLAP), type I collagen (COL1), bone sialoprotein (IBSP), osteonectin (SPARC) and osteopontin (SPP1) was analyzed by real-time quantitative polymerase chain reaction (qPCR). Total RNA was extracted from the cell cultures using the Maxwell RSC simplyRNA tissue kit (Promega, Madison, WI, USA) according to the manufacturer’s protocol. RNA concentration and purity were determined using a Nanodrop spectrophotometer (Nanodrop 1000, Thermo Scientific, ThermoFisher Scientific). Reverse transcription was performed with 500 ng total RNA using the iScript cDNA synthesis kit (Biorad, Hercules, CA, USA), according to the manufacturer’s instructions. The mRNA levels were assayed in triplicate in CFX384 arrays (BioRad) using 5 µL of iTaq Universal SYBR Green Supermix (BioRad), 0.5 µL of PrimePCR Assays (BioRad) and 20 ng cDNA in a total volume of 10 µL. The PCR amplification was performed as follows: initial heating at 95 °C for 3 min, followed by 40 cycles at 95 °C for 10 s, 60 °C for 30 s, and a final melt curve from 65 °C to 95 °C, in 0.5° increment each 5 s in a C1000 Touch Thermal Cycler (BioRad). Expression values were obtained from cycle quantification (Cq) values determined with the BioRad CFX Maestro™ Software. The target gene levels are expressed as a relative value, the ratio of the target gene expression to that of the reference TATA-box binding protein (TBP) and hypoxantine phosphoribosyltranferase (HPRT1) genes. The relative gene expression was calculated as 2-△Cq. Validated PrimePCR SYBR Green Assays (BioRad) for ALPL (qHsaCID0010031), BGLAP (qHsaCED0038437), COL1 (qHsaCED0043248), IBSP (qHsaCED0002933), SPARC (qHsaCID0010332), SPP1 (qHsaCID0012060), TBP (qHsaCID0007122) and HPRT1 (qHsaCID0016375) were used.

### 2.10. Mineralization Assay

Osteoblasts differentiation was studied through the detection of calcium deposits, a sign of extracellular matrix (ECM) mineralization. Calcium deposition was determined by alizarin red S (ARS). Cells were first rinsed twice in cold PBS, fixed in 4% paraformaldehyde in PBS for 30 min at RT and then washed twice with distilled water. An incubation with 2% ARS in distilled water at pH 4.2 was used to stain the cells for 45 min at RT in the dark. Finally, samples were washed four times with distilled water and visualized in an Olympus IX71 inverted microscope. To measure absorbance, the incorporated ARS dye was extracted from the cell cultures with 10% cetylpiridinium chloride (CPC) in 10 mM sodium phosphate at pH 7 for 15 min on a shaker at RT. The extracted stain was transferred to a 96-well plate and the absorbance at 540 nm was measured using a Victor III microplate scanner (Perkin Elmer, Waltham, MA, USA) and normalized using Alamar Blue results.

### 2.11. Statistical Analysis

All quantitative data were analyzed with GraphPad Prism 6 (GraphPad Software Inc., San Diego, CA, USA) and presented as the mean ± standard error of the mean. Statistical differences were assessed by two-way analysis of variance (ANOVA). A value of *p* < 0.05 was considered to be significant. Significance is represented in the figures using an alphabetical superscript system on top of the columns. Values with different alphabetical superscripts are significantly different (*p* = 0.05), whereas values with the same alphabetical superscripts are not significantly different.

## 3. Results

### 3.1. Structural Characterization

**Figure 1:**
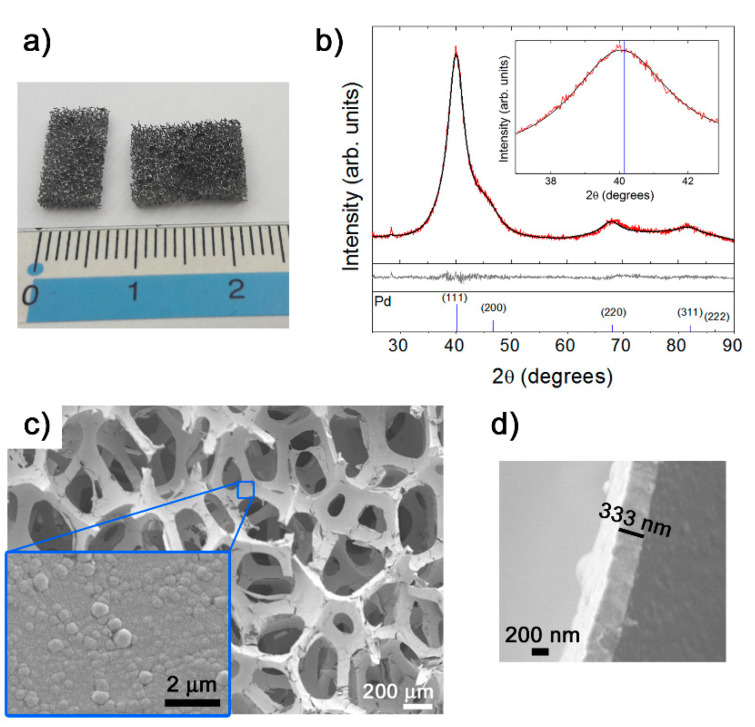
(**a**) Picture of pieces of Pd-coated polyurethane (PU) foam and (**b**) experimental XRD pattern (red line). The calculated fit using the HighScore Plus software is also shown (black line) (the peak at 29° belongs to the sample holder). The difference between the experimental and calculated profiles and the reference pattern for fcc-Pd depicted in the middle and bottom panels, respectively. SEM images of (**c**) Pd-coated PU foam and on-top morphology of the PdP layer (inset) and (**d**) cross-section depicting the thickness of the layer.

A picture showing the metal-like appearance of the PU foam after electroless Pd metallization can be seen in Figure 1a. The corresponding experimental XRD pattern is presented in Figure 1b (red line). The main peaks from face-centered cubic (fcc) palladium (space group, Fm3m, reference pattern #00-005-0681) are indicated at the bottom of the figure. The observed peaks in the Pd-coated PU foam pattern agree with the tabulated fcc-Pd phase, although they are slightly shifted to lower angles with respect to the tabulated position (see the detail of the (111) peak in the inset of Figure 1b). A cell parameter of 3.9275 Å, a crystallite size of 27.9 Å ± 3.1 Å and a microstrain of 0.019% are calculated using the Rietveld fit (black line, Figure 1). The calculated larger cell parameter compared to the tabulated one (a = 3.8898 Å) can be attributed to the incorporation of P, which has a smaller atomic radius than Pd [31], into interstitial positions of the fcc-Pd lattice. This cell expansion has been observed in other electroless metallic coatings like NiP [32]. The PU did not give any detectable response, which means that it is basically amorphous.

Figure 1c shows a low magnification SEM image of the Pd-coated PU foam in which the open-cell architecture provided by the PU can be observed. Pores have dimensions in the macroscale (roughly around 200–400 μm) and are circular or elliptic. Importantly, the PdP coating conformally coats the PU walls. An SEM image of the on-top nodular-type morphology of the metallic coating is shown in the inset. The thickness of this layer is around 333 nm (Figure 1d).

### 3.2. Cell Proliferation

**Figure 2:**
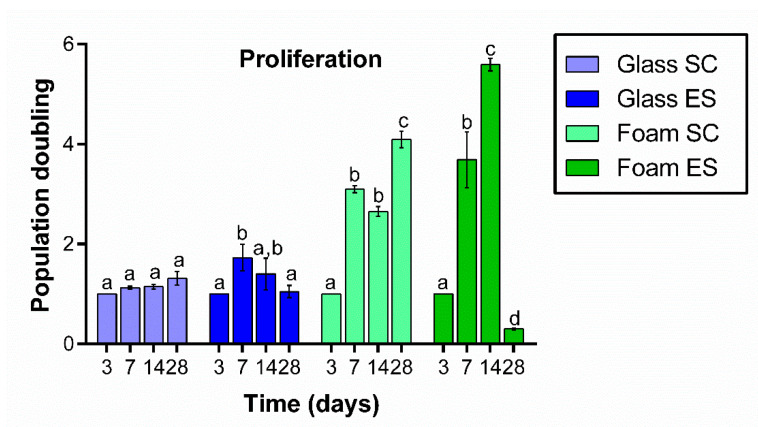
Proliferation of Saos-2 cells grown on control glass coverslips and on Pd-coated PU foams at 3, 7, 14 and 28 days in culture in standard conditions (SC) or under electrical stimulation (ES). Results are normalized by day 3. Different superscripts on top of the columns denote significant differences (*p* < 0.05) among different time-points of cells grown on the same materials and under the same conditions.

The proliferation of Saos-2 cells grown on Pd-coated PU foams under SC or ES was quantified after 3, 7, 14 and 28 days of seeding and normalized by day 3. As it can be seen in Figure 2, the number of cells significantly increased between days 3 and 14, especially in the case of cells cultured under ES. Cell number continued to increase after 28 days of culture under SC, but a large significant drop was observed in cultures under ES.

When the same number of cells were seeded on control glass coverslips, proliferation was not significant under SC at any time-point analyzed. Under ES conditions, the number of cells increased significantly on day 7, stabilized on day 14 and then slightly decreased on day 28.

### 3.3. Cell Morphology and Adhesion

**Figure 3:**
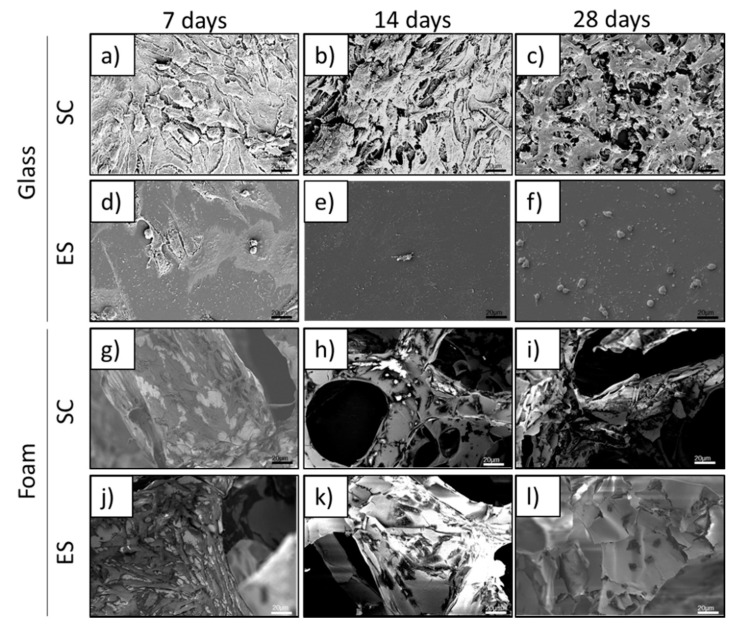
SEM images of human Saos-2 cells cultured for 7, 14 and 28 days under standard conditions (SC) or electrical stimulation (ES) on either glass coverslips (**a**–**f**) or Pd-coated PU foams (**g**–**l**).

SEM analyses of osteoblasts grown on Pd-coated PU foams or on glass coverslips were carried out at 7, 14 and 28 days (Figure 3). Cells grown on foams for 7 and 14 days under both SC and ES were well spread and showed a flattened appearance (Figure 3g,h,j,k), indicating that they were attached to the surface. In agreement with the cell proliferation results on day 28, good morphology and attachment was still observed for cells grown under SC (Figure 3i), but under ES only a few cells remained attached to the foam (Figure 3l). These cells were smaller and showed rounded morphology, suggesting that they were starting to die.

SEM images of cells grown on control glass coverslips under SC (Figure 3a–c) revealed that cultures had already reached confluence on day 7. These cells showed a flat and elongated morphology, compatible with good attachment to the surface. Under ES, the density of osteoblasts was extremely low on glass coverslips on days 14 and 28, with cells presenting a round morphology and poorly attached to the surface (Figure 3e,f).

**Figure 4:**
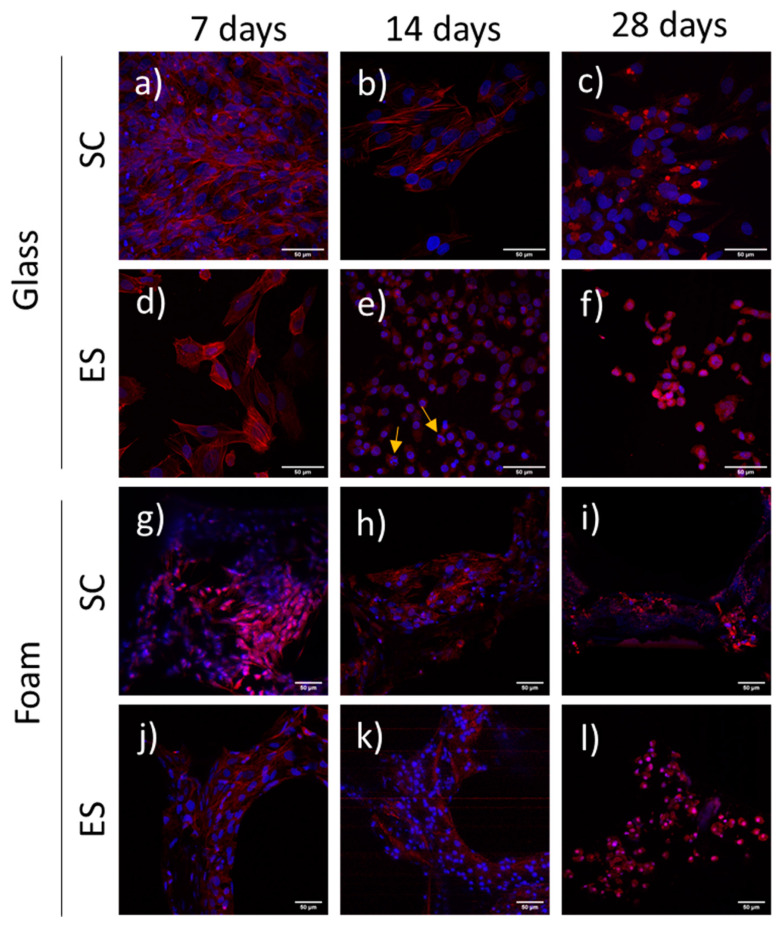
Actin distribution in human Saos-2 cells cultured for 7, 14 and 28 days under standard conditions (SC) or electrical stimulation (ES) on either glass coverslips (**a**–**f**) or Pd-coated PU foams (**g**–**l**). Stress fibers (red) and nuclei (blue) can be observed. Yellow arrows point to apoptotic nuclei. Pd-coated PU foams images (**g**–**l**) were obtained at less augmentation and digitally augmented due to the nature of the foam material.

To further evaluate cell adhesion, actin filaments, the constituents of stress fibers, were labelled and analysed at 7, 14 and 28 days (Figure 4). Confocal image acquisition of cells grown on foams required a lower magnification than for cells grown on glass coverslips, because of the nature of the foam material. The 3D structure of the Pd-coated PU foams was very difficult to focus under CLSM at high magnification due to the pore topology, non-uniform structure and high light reflection. Cells grown on foams under both SC and ES presented long actin fibers crossing the cells at 7 and 14 days (Figure 4g,h,j,k). On day 28, cells under SC still displayed defined stress fibers (Figure 4i), but cells under ES were smaller, rounded and with non-structured stress fibers (Figure 4l).

Control cells grown on glass coverslips under SC presented well-defined stress fibers, some of them crossing the cells from end to end, on days 7 and 14 (Figure 4a,b). However, on day 28 the cells had rounded and stress fibers were no longer visible (Figure 4c). In the case of cells grown under ES, long and defined stress fibers were only detected on day 7 of culture (Figure 4d). On days 14 and 28, cells were less spread and stress fibers were shorter and poorly structured (Figure 4e,f). Some apoptotic-looking nuclei could be observed on day 14 (Figure 4e).

### 3.4. Expression of Osteogenic Markers

**Figure 5:**
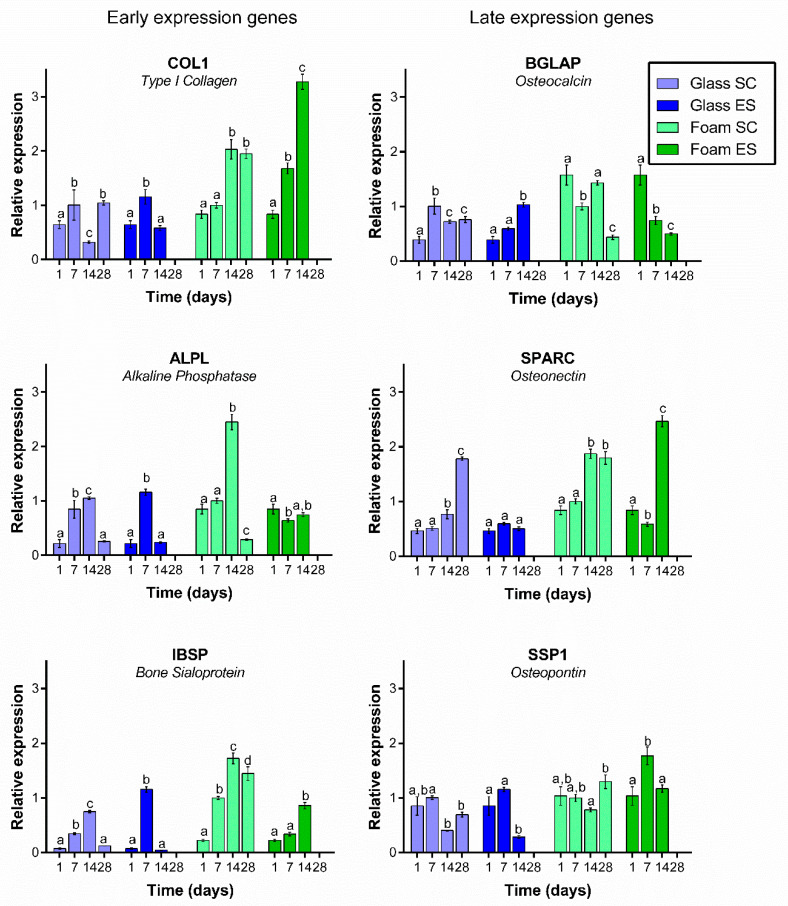
Relative expression of osteoblast differentiation markers COL1, ALP, BGLAP, IBSP, SPARC and SPP1 in Saos-2 cells cultured on glass coverslips or Pd-coated PU foams under standard conditions (SC) or electrical stimulation (ES) for 1, 7 14 or 28 days. Different superscripts on top of the columns denote significant differences (*p* < 0.05) among different time-points of cells grown on the same materials and under the same conditions.

The expression of six specific osteoblast differentiation markers (ALPL, BGLAP, COL1, IBSP, SPARC, SPP1) was evaluated after 1, 7, 14 and 28 days in culture on Pd-coated PU foams and on glass surfaces in both conditions (Figure 5). ES results on day 1 are the same as SC results because stimulation started on day 3. No RNA could be extracted from ES cells at 28 days because cell numbers were too low.

In general, expression of early osteogenic markers significantly increased along time in culture in cells grown in foams under both conditions. The only exception was ALPL, which maintained its expression level in cells under ES, or increased and then decreased in cells under SC. The expression of these early genes followed a similar pattern in control cells grown on glass coverslips under SC (except for IBSP), but an opposite pattern in control cells under ES. In the latter, all three genes showed a significant decrease from day 7 to day 14.

Expression of late osteogenic markers in cells cultured on Pd-coated PU foams did not follow a clear trend. Under SC, BGLAP expression was decreased on day 28 when compared with day 1, whereas SPARC increased and SSP1 was maintained. A similar pattern occurred in cells under ES, except that SSP1 expression was decreased in this case. In control cells grown on glass coverslips, expression of BGLAP increased over time both under SC and ES, SPARC expression increased under SC but was maintained under ES, and SSP1 expression was maintained under SC but decreased under ES.

### 3.5. Calcium Deposition

**Figure 6:**
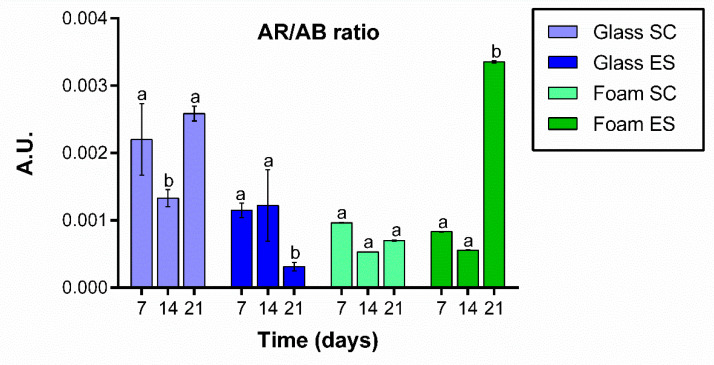
Extracellular matrix mineralization. Calcium deposit quantification of Saos-2 cells grown on glass coverslips or Pd-coated PU foams surfaces under standard conditions (SC) or electrical stimulation (ES) at 7, 14 and 21 days of culture. Different superscripts on top of the columns denote significant differences (*p* < 0.05) among different time-points of cells grown on the same materials and under the same conditions.

Cultures were stained with Alizarin Red S on days 7, 14, and 21. The test was carried out at 21 days instead of 28 to be able to quantify the calcium secretion before the decrease in cell viability seen at 28 days. To normalize the calcium deposition with cell population, CPC extracts were normalized to Alamar blue results. As can be seen, calcium deposition secreted by cells grown on foams under SC did not significantly vary along time. Nevertheless, cells grown on foams under ES secreted significantly higher amounts of calcium deposits at day 21 of culture (Figure 6).

On day 7, cells grown on glass coverslips under SC secreted a significantly higher number of calcium deposits than cells from the other groups at same day. Cells grown on glass coverslips under ES secreted similar amounts of calcium deposits at days 7 and 14 but, contrary to cells grown on foams under ES, a significant decrease in calcium secretion was detected on day 21.

## 4. Discussion

The objective of this study was to assess potential differences in cell behavior, regarding proliferation and differentiation, in osteoblasts grown on highly porous, electrically conductive 3D scaffolds in standard conditions or under electrical stimulation. Specifically, we exploited the concept of bipolar electrochemistry [25], in which the bipolar electrode (i.e., the Pd-coated PU foam) becomes polarized even in the absence of direct ohmic contact with the Pd layer. Pd was selected owing to its noble character to avoid eventual electrodissolution upon voltage application. The culture medium served as the electrolyte solution and upon voltage application a potential of opposite polarity was induced in the Pd-coated PU foam [33]. To this aim, we analyzed the viability, adhesion, proliferation and differentiation of the osteoblastic cell type Saos-2 on Pd-coated PU foams up to 28 days in culture in presence of an electrical wave following arbitrary sinusoid function of 2.5 V (100 mV/mm) amplitude and 200 Hz frequency for 1 h each day, as Qi et al. found out that it was the most suitable frequency for enhancing osteoblasts’ growth [34]. Saos-2 cells were used because it has been described that they behave in the same way as primary osteoblast cells in culture [35]. The open-cell PU enabled cells to freely grow onto their Pd metallized walls throughout the entire 3D structure without interference while having access to the nutrients. Both SEM and XRD analyses of the material confirmed the successful conformal metallization of the PU foam with PdP by electroless plating.

Cells grown on Pd-coated PU foams under ES presented higher proliferation than cells grown on foams without ES at days 7 and 14, even though at day 28 they started to die. These results are in accordance with previous findings demonstrating that the application of specific ES parameters through conductive polymer substrates can modulate important osteoblast markers [26]. However, it has also been described that a stimulation regime comprising high voltage (>300 mV), several hours of daily stimulation or stimulation for more than 21 days can negatively affect proliferation [2,36,37,38]. This could explain the low proliferation values obtained in the present work after 28 days under ES.

It should also be noted that foams provide a larger surface area than glass coverslips, which is beneficial for cell growth and proliferation. The pore size of the Pd-coated PU foam is between 200 and 400 μm, which is of the order of the pore size of polymeric scaffolds previously reported in the literature. For example, Zhang et al. prepared polypyrrole-based scaffolds with a pore size of 240 ± 76 μm [2]. Compared to this study, however, the pores of the Pd-coated PU foam are more interconnected. Importantly, pore sizes in the range of 200–400 μm are considered optimum for nutrient transport and ingrowth of bone tissue [39]. While control cells reached saturation by day 7, as it can also be seen in the SEM analyses, proliferation could be detected on foams until day 14 of ES or up to 28 days in non-stimulated ones, demonstrating the suitability of these foams as scaffolds for osteoblast proliferation.

Surface topography can modulate morphology and cell adhesion, as well as interactions between cells and the material. Cells grown on foams subject to SC were well adhered, with a flat appearance and a typical actin cytoskeleton distribution, indicating that this material was optimal for osteoblast growth; by 28 days the cells were filling the entire structure. By contrast, cells electrically stimulated for 28 days appeared rounded and not well adhered, a sign of cell death onset. When cells begin to die, they detach from the surface and eventually die [40]. These observations are in agreement with the aforementioned abrupt decrease in the proliferation of cells cultured for 28 days on foams under ES. The formation of reactive oxygen species in the culture medium, if any, cannot explain cell death as their concentration should be similar, irrespective of the bipolar electrode used (glass coverslip and Pd-coated PU foam).

Once it was demonstrated that daily ES of foams increased the proliferation of osteoblast at least up to 14 days, we decided to analyze the expression of six different genes related with osteoblast differentiation. Osteoblast differentiation is usually divided in two stages: an early stage, when the extracellular matrix begins to mature, and a late stage, when the extracellular matrix mineralizes [41,42,43]. The length of each period is not clear and varies among reports, but the extend of the early stage is considered by most authors to be between 7 and 14 days, and at approximatively 14 days the late stage begins [41,42,43]. Each of these two stages, maturation and mineralization, are regulated by several genes. Among the genes expressed during osteoblast differentiation we have selected six different genes: COL1, ALPL and IBSP, generally considered as early expressed genes, and BGLAP, SPARC and SPP1, considered late expressed genes.

ALPL gene encodes for alkaline phosphatase protein and, generally, it is accepted that this gene is expressed through proliferation and early extracellular matrix maturation, that is, between 7–14 days [41,43]. However, some authors have also reported ALPL expression in late stages [44]. In the present study, ALPL was upregulated in cells grown on foams under SC for 14 days, decreasing abruptly by day 28. However, under ES, the expression of ALPL was similar at all time-points analysed. ALPL is a controversial gene because its expression is very variable depending on the material used, the voltage applied or the time under culture. For instance, Zhang and co-workers [2] found an increase in ALP activity in stem cells (AD-MSC) cultured in an electrically conductive polypyrrole/polycaprolactone scaffold subjected to 200 μA of direct current for 4 h/day during 21 days.

COL1 gene encodes for type I collagen and, according to several authors [41,42], its maximum expression is during proliferation and decreases during extracellular matrix maturation. However, some other authors consider COL1 as a late stage expression gene [43]. In our work, the expected pattern was clear on cells grown on glass, electrically stimulated or not, where a decrease at 14 days was observed, but not on Pd-coated PU foams where an increased level of COL1 expression was still present at day 14. A similar behavior has been reported by other authors [41] testing different kinds of materials and levels of roughness. ES appears to increase COL1 expression compared with SC. This increase could be related to the proliferation observed at day 14 on ES foams. Collagen I is a protein involved in extracellular matrix formation and if proliferation is still active at 14 days, collagen is still necessary to build the extracellular matrix. Another gene expressed at early extracellular matrix mineralization stages is IBSP, encoding Bone Sialoprotein. The pattern of IBSP expression observed in our study was very similar to that of COL1. IBSP was still upregulated at day 14 in cells grown on ES or SC foams. Bone sialoprotein seems to act as a nucleus for the formation of the apatite crystals, and together with collagen will form the extracellular matrix. This gene has also been found up-regulated in osteoblasts grown on rough material [41,45].

BGLAP, SPARC and SPP1 genes encode osteocalcin (OC or OCN), osteonectin (ON) and osteopontin (OPN) proteins, respectively. The three proteins are Ca^2+^-binding proteins and, together with calcium, they are responsible for the extracellular matrix mineralization. When analyzing their expression in osteoblast grown on foams, a different behavior was observed depending on the gene and on whether the foam had been ES or not. It is difficult to explain the decrease in BGLAP expression in osteoblast grown on ES foams from day 7, as this gene is supposed to have its maximum expression between 7 and 14 days [42]. In osteoblasts grown under SC, this decrease was indeed observed at 28 days. A completely different expression pattern was observed in osteoblast grown on ES glass, where BGLAP expression increased up to day 14. Liu and co-workers [14] also reported a decrease in the expression of BGLAP in MC3T3 cells cultured on Ppy IDE with 1 h daily stimulation (15 mV) for 7 and 14 days, which is in agreement with our results. By contrast, SPARC expression was upregulated in osteoblast grown on both ES and SC foams, with higher expression under ES. Osteonectin, the protein encoded by SPARC, has an affinity for collagen, and collagen was highly expressed in osteoblasts under ES. The expression of this gene follows the pattern described in other works [41]. Finally, SSP1 gene was down-regulated in osteoblast grown under ES in both Pd-coated PU foams or glass surfaces at 14 days. Liu et al. [14] also reported a decrease in the expression of this gene at 14 days under the conditions described above. So, when considering the three genes corresponding to the mineralization stage, we found that SPARC and SPP1 were more highly expressed in osteoblast grown on ES foams than in non-stimulated ones. When comparing the behavior of these three genes in osteoblasts grown on glass, we can conclude that expression was higher or similar in Pd-coated PU foams than in glass surfaces. Due to the decreased cell numbers at 28 days, it was not possible to obtain enough mRNA to analyze the expression of these genes at this time-point.

When we quantified calcium release in osteoblasts grown on glass or foams with or without ES for up to 21 days, we found that mineralization was higher in cells grown on foams under ES than SC, which corroborates the fact that ES favors the differentiation of osteoblasts for at least up to 21 days.

## 5. Conclusions

In summary, our results show that electroless Pd-coated PU foams seem to be good candidates for osteoblast scaffolds because they sustain proliferation for up to 28 days under SC and promote the expression of several genes related to extracellular matrix formation and mineralization, when compared with glass surfaces. However, when osteoblast grown on foams or glass are electrically stimulated, proliferation stops, and cells die after 28 days of daily stimulation. Electrical stimulation seems to enhance expression of early expressed genes related to extracellular matrix formation (COL1 and IBSP), but its effects on late expressed genes related to matrix mineralization are gene-dependent.

## Data Availability

The data presented in this study are available on request from the corresponding author. The data are not publicly available due to their use in further studies.

## References

[1] Narkevica I., Stipniece L., Jakobsons E., Cakstina I., Ozolins J. (2017). Electrically active and 3D porous TiO_2_-x ceramic scaffolds for bone tissue regeneration. J. Eur. Ceram. Soc..

[2] Zhang J., Li M., Kang E.T., Neoh K.G. (2016). Electrical stimulation of adipose-derived mesenchymal stem cells in conductive scaffolds and the roles of voltage-gated ion channels. Acta Biomater..

[3] Leppik L., Oliveira K.M.C., Bhavsar M., Barker J.H. (2020). Electrical stimulation in bone tissue engineering treatments. Eur. J. Trauma Emerg. Surg..

[4] Lafage-Proust M., Roche B., Langer M., Cleret D., Bossche A.V., Olivier T., Vico L. (2015). Assessment of bone vascularization and its role in bone remodeling. BoneKEy Rep..

[5] Loh Q.L., Choong C. (2013). Three-dimensional scaffolds for tissue engineering applications: Role of porosity and pore size. Tissue Eng. Part B Rev..

[6] Mesquita-Guimarães J., Ramos L., Detsch R., Henriques B., Fredel M.C., Silva F., Boccaccini A.R. (2019). Evaluation of in vitro properties of 3D micro-macro porous zirconia scaffolds coated with 58S bioactive glass using MG-63 osteoblast-like cells. J. Eur. Ceram. Soc..

[7] Metz C., Duda G.N., Checa S. (2020). Towards multi-dynamic mechano-biological optimization of 3D-printed scaffolds to foster bone regeneration. Acta Biomater..

[8] Parai R., Bandyopadhyay-Ghosh S. (2019). Engineered bio-nanocomposite magnesium scaffold for bone tissue regeneration. J. Mech. Behav. Biomed. Mater..

[9] Tayebi L., Shahini A., Yazdimamaghani M., Walker K.J., Eastman M.A., Hatami-Marbini H., Smith B.J., Ricci J.L., Madihally S.V., Vashaee D. (2013). 3D conductive nanocomposite scaffold for bone tissue engineering. Int. J. Nanomed..

[10] Jing W., Huang Y., Wei P., Cai Q., Yang X., Zhong W. (2019). Roles of electrical stimulation in promoting osteogenic differentiation of BMSCs on conductive fibers. J. Biomed. Mater. Res. Part A.

[11] Hardy J.G., Sukhavasi R.C., Aguilar D., Villancio-Wolter M.K., Mouser D.J., Geissler S.A., Nguy L., Chow J.K., Kaplan D.L., Schmidt C.E. (2015). Electrical stimulation of human mesenchymal stem cells on biomineralized conducting polymers enhances their differentiation towards osteogenic outcomes. J. Mater. Chem. B.

[12] Pelto J., Björninen M., Pälli A., Talvitie E., Hyttinen J., Mannerström B., Seppanen R.S., Kellomäki M., Miettinen S., Haimi S. (2013). Novel polypyrrole-coated polylactide scaffolds enhance adipose stem cell proliferation and early osteogenic differentiation. Tissue Eng. Part A.

[13] Nguyen H.T., Sapp S., Wei C., Chow J.K., Nguyen A., Coursen J., Luebben S., Chang E., Ross R., Schmidt C.E. (2013). Electric field stimulation through a biodegradable polypyrrole- co -polycaprolactone substrate enhances neural cell growth. J. Biomed. Mater. Res. Part A.

[14] Liu Z., Dong L., Cheng K., Luo Z., Weng W. (2018). Charge injection based electrical stimulation on polypyrrole planar electrodes to regulate cellular osteogenic differentiation †. RSC Adv..

[15] Jin G., Kim G. (2013). The effect of sinusoidal AC electric stimulation of 3D PCL/CNT and PCL/β-TCP based bio-composites on cellular activities for bone tissue regeneration. J. Mater. Chem. B.

[16] Vila M., Cicuéndez M., Sánchez-Marcos J., Fal-Miyar V., Manzano M., Prieto C., Vallet-Regi M. (2013). Electrical stimuli to increase cell proliferation on carbon nanotubes/mesoporous silica composites for drug delivery. J. Biomed. Mater. Res. Part A.

[17] Mata D., Oliveira F.J., Neto M.A., Belmonte M., Bastos A.C., Lopes M.A., Gomes P.S., Fernandes M.H., Silva R.F. (2015). Smart electroconductive bioactive ceramics to promote in situ electrostimulation of bone. J. Mater. Chem. B.

[18] Rajabi A.H., Jaffe M., Arinzeh T.L. (2015). Piezoelectric materials for tissue regeneration: A review. Acta Biomater..

[19] Murillo G., Blanquer A., Vargas-Estevez C., Barrios L., Ibáñez E., Nogués C., Esteve J. (2017). Electromechanical Nanogenerator–Cell Interaction Modulates Cell Activity. Adv. Mater..

[20] Marino A., Genchi G.G., Mattoli V., Ciofani G. (2017). Piezoelectric nanotransducers: The future of neural stimulation. Nano Today.

[21] Timin A.S., Muslimov A.R., Zyuzin M.V., Peltek O.O., Karpov T.E., Sergeev I.S., Dotsenko A.I., Goncharenko A.A., Yolshin N.D., Sinelnik A. (2018). Multifunctional Scaffolds with Improved Antimicrobial Properties and Osteogenicity Based on Piezoelectric Electrospun Fibers Decorated with Bioactive Composite Microcapsules. ACS Appl. Mater. Interfaces.

[22] Chernozem R.V., Surmeneva M.A., Shkarina S.N., Loza K., Epple M., Ulbricht M., Cecilia A., Krause B., Baumbach T., Abalymov A.A. (2019). Piezoelectric 3-D Fibrous Poly(3-hydroxybutyrate)-Based Scaffolds Ultrasound-Mineralized with Calcium Carbonate for Bone Tissue Engineering: Inorganic Phase Formation, Osteoblast Cell Adhesion, and Proliferation. ACS Appl. Mater. Interfaces.

[23] Park J., Mazare A., Schneider H., Von Der Mark K., Fischer M.J.M., Schmuki P. (2016). Electric Field-Induced Osteogenic Differentiation on TiO_2_ Nanotubular Layer. Tissue Eng. Part C Methods.

[24] Woo D.G., Shim M.S., Park J.S., Yang H.N., Lee D.R., Park K.H. (2009). The effect of electrical stimulation on the differentiation of hESCs adhered onto fibronectin-coated gold nanoparticles. Biomaterials.

[25] Fosdick S.E., Knust K.N., Scida K., Crooks R.M. (2013). Bipolar electrochemistry. Angew. Chem. Int. Ed..

[26] Qin C., Yue Z., Chao Y., Forster R.J., Maolmhuaidh F.Ó., Huang X.-F., Beirne S., Wallace G.G., Chen J. (2020). Data on the bipolar electroactive conducting polymers for wireless cell stimulation. Data Brief.

[27] Wataha J.C., Shor K. (2010). Palladium alloys for biomedical devices. Expert Rev. Med. Devices.

[28] Woodward B. (2012). Palladium in temporary and permanently implantable medical devices. Platin. Met. Rev..

[29] Cowley A., Woodward B. (2011). A healthy future: Platinum in medical applications platinum group metals enhance the quality of life of the global population. Platin. Metals Rev..

[30] Leppik L., Bhavsar M.B., Oliveira K.M.C., Eischen-Loges M., Mobini S., Barker J.H. (2019). Construction and Use of an Electrical Stimulation Chamber for Enhancing Osteogenic Differentiation in Mesenchymal Stem/Stromal Cells In Vitro. J. Vis. Exp..

[31] Ghosh D., Biswas R. (2002). Theoretical Calculation of Absolute Radii of Atoms and Ions. Part 1. The Atomic Radii. Int. J. Mol. Sci..

[32] Salicio-Paz A., Grande H., Pellicer E., Sort J., Fornell J., Offoiach R., Lekka M., García-Lecina E. (2019). Monolayered versus multilayered electroless NiP coatings: Impact of the plating approach on the microstructure, mechanical and corrosion properties of the coatings. Surf. Coat. Technol..

[33] Rajnicek A.M., Zhao Z., Moral-Vico J., Cruz A.M., McCaig C.D., Casañ-Pastor N. (2018). Controlling Nerve Growth with an Electric Field Induced Indirectly in Transparent Conductive Substrate Materials. Adv. Healthc. Mater..

[34] Qi Z., Xia P., Pan S., Zheng S., Fu C., Chang Y., Ma Y., Wang J., Yang X.-Y. (2018). Combined treatment with electrical stimulation and insulin-like growth factor-1 promotes bone regeneration in vitro. PLoS ONE.

[35] Czekanska E.M., Stoddart M.J., Richards R.G., Hayes J.S. (2012). In search of an osteoblast cell model for in vitro research. Eur. Cells Mater..

[36] Meng S., Rouabhia M., Zhang Z. (2013). Electrical stimulation modulates osteoblast proliferation and bone protein production through heparin-bioactivated conductive scaffolds. Bioelectromagnetics.

[37] Suryani L., Too J.H., Hassanbhai A.M., Wen F., Lin D.J., Yu N., Teoh S.-H. (2019). Effects of electromagnetic field on proliferation, differentiation, and mineralization of MC3T3 Cells. Tissue Eng. Part C Methods.

[38] Zhu S., Jing W., Hu X., Huang Z., Cai Q., Ao Y., Yang X. (2017). Time-dependent effect of electrical stimulation on osteogenic differentiation of bone mesenchymal stromal cells cultured on conductive nanofibers. J. Biomed. Mater. Res. Part A.

[39] Bose S., Roy M., Bandyopadhyay A. (2012). Recent advances in bone tissue engineering scaffolds. Trends Biotechnol..

[40] Frisch S.M., Screaton R.A. (2001). Anoikis mechanisms. Curr. Opin. Cell Biol..

[41] Setzer B., Bächle M., Metzger M.C., Kohal R.J. (2009). The gene-expression and phenotypic response of hFOB 1.19 osteoblasts to surface-modified titanium and zirconia. Biomaterials.

[42] Neve A., Corrado A., Cantatore F.P. (2010). Osteoblast physiology in normal and pathological conditions. Cell Tissue Res..

[43] Mizerska-Kowalska M., Sławinska-Brych A., Kaławaj K., Zurek A., Pawinska B., Rzeski W., Zdzisińska B. (2019). Betulin promotes differentiation of human osteoblasts in vitro and exerts an osteoinductive effect on the HfOB 1.19 cell line through activation of JNK, ERK1/2, and mTOR kinases. Molecules.

[44] Wiesmann H.P., Hartig M., Stratmann U., Meyer U., Joos U. (2001). Electrical stimulation influences mineral formation of osteoblast-like cells in vitro. Biochim. Biophys. Acta-Mol. Cell Res..

[45] Ogawa T., Nishimura I. (2003). Different bone integration profiles of turned and acid-etched implants associated with modulated expression of extracellular matrix genes. Int. J. Oral Maxillofac. Implant..

